# Disproportionality Analysis of the Five Most Widespread Neurological Effects of COVID-19 Vaccines from 2021 to 2023: Insights from EudraVigilance

**DOI:** 10.3390/ph18050636

**Published:** 2025-04-27

**Authors:** Arturo Gómez López de las Huertas, Stefan Stewart, Mikel Urroz Elizalde, Javier Guijarro-Eguinoa, Enrique Seco-Meseguer, Elena Diago-Sempere, María Jiménez González, Antonio J. Carcas-Sansuan, Alberto M. Borobia Pérez, Elena Ramírez

**Affiliations:** 1 Clinical Pharmacology Department, La Paz University Hospital-IdiPAZ, School of Medicine, Autonomous University of Madrid, 28046 Madrid, Spain; stefanmark.stewart@salud.madrid.org (S.S.); mikel.urroz@salud.madrid.org (M.U.E.); franciscojavier.guijarro@salud.madrid.org (J.G.-E.); enrique.seco@salud.madrid.org (E.S.-M.); elenaisabel.diago@salud.madrid.org (E.D.-S.); antonio.carcas@uam.es (A.J.C.-S.); alberto.borobia@salud.madrid.org (A.M.B.P.); 2 Clinical Trials Unit, La Paz University Hospital-IdiPAZ, 28046 Madrid, Spain; maria.jimenez.gonzalez@salud.madrid.org

**Keywords:** pharmacovigilance, EudraVigilance, disproportionality, vaccines, COVID-19

## Abstract

**Background/Objectives:** Post-market surveillance of COVID-19 vaccines is vital. This study analyzed EudraVigilance data (Jan 2021–Dec 2023) to detect potential safety signals linking COVID-19 vaccines and specific neurological adverse events (aseptic meningitis, Guillain–Barré syndrome, polyradiculoneuropathies, multiple sclerosis, transverse myelitis, neuromyelitis optica). It also explored the impact of non-healthcare professional reports on disproportionality analysis. **Methods:** EudraVigilance reports were analyzed to quantify neurological events for 5 COVID-19 vaccines and 47 comparators. Disproportionality was assessed using the Proportional Reporting Ratio (PRR). Spearman’s correlation (SCC) was used to examine the impact of non-healthcare professional reports on PRR. **Results:** An analysis of 4,159,820 COVID-19 vaccine and 114,025 comparator reports showed a reporting decline over time. A higher proportion of adverse drug event reports were submitted by non-healthcare professionals for COVID-19 vaccines compared to control vaccines, a trend observed consistently across 2021 (57.3% vs. 33%, *p* < 0.001), 2022 (59.4% vs. 36.5%, *p* = 0.001), and 2023 (42% vs. 24.36%, *p* = 0.006). In 2023, significant signals (PRR ≥ 2) were found between Jcovden© and polyradiculoneuropathy (PRR 5.4, IC 95% 3.98–7.32), multiple sclerosis (PRR 2.72, IC 95% (1.08–6.87), transverse myelitis (PRR 4.68, IC 95% 1.02–21.35) and neuromyelitis optica (PRR 7.79, IC 95% 3.5–17.37). In addition, both Spikevax© and Comirnaty© showed significant signals with multiple sclerosis (PRR 2.50, IC 95% 1.70–3.68, and PRR 2.33, IC 95% 1.68–3.24) and transverse myelitis (PRR 3.50, IC 95% 1.66–7.50 and PRR 3.58, IC 95% 1.85–6.93). A significant negative correlation between the proportion of reports from non-healthcare professionals and the case/no-case ratio was found (SCC = −0.4683, *p* = 0.009). **Conclusions:** While some significant signals emerged in 2023, the combined three-year data showed no vaccine exceeding the PRR threshold of 2. High-quality data and bias mitigation strategies are crucial for accurate PRR estimation in pharmacovigilance and public health.

## 1. Introduction

The COVID-19 public health emergency of international concern ended in May 2023 [1], after originally being declared by the World Health Organization (WHO) in January 2020 [2]. Despite this, COVID-19 remains a global health threat as it still places a burden on healthcare systems and individuals whose immunity has decreased with time [3]. As of June 2024, 776,007,137 cases and 7,059,612 deaths have been reported worldwide since the beginning of the COVID-19 pandemic [4]. Considering that approximately 5,700,000 deaths occurred in the first two years of the pandemic [4], we can see that the trend has declined considerably, largely due to both the passive and active immunization of the population.

### 1.1. COVID-19 Vaccines

The first COVID-19 vaccine authorized by the European Medicines Agency (EMA) was the Comirnaty© mRNA vaccine marketed by Pfizer-BioNTech [5], which received approval on 21 December 2020. Shortly after, three more vaccines were approved: the Spikevax© mRNA vaccine by Moderna [6], the Vaxzevria© adenovirus vaccine by AstraZeneca [7] and the Jcovden© adenovirus vaccine by Janssen [8]. The Nuvaxovid© vaccine by Novavax [9] followed on 20 December 2021, and the Bimervax© vaccine by HIPRA [10] on 30 March 2023. However, marketing authorization for Jcovden and Vaxzevria was later withdrawn at the request of their manufacturers.

As of 31 December 2023 (latest WHO report cut-off date available to date), approximately 13,650,000,000 vaccine doses had been administered worldwide, with roughly 67% of the population having received a complete primary vaccination series [11]. While reinfections occur, particularly during winter months, real-world data demonstrate the vaccines’ effectiveness in reducing hospitalizations and improving overall survival [12,13]. However, the clinical benefit of all drugs is always contingent on the occurrence of adverse drug effects (AEs), and COVID-19 vaccines are no exception.

#### COVID-19 Vaccines’ Safety Profile

Although the safety profile of COVID-19 vaccines is generally acceptable, this does not mean that they are risk-free. In the pivotal clinical trials of the first four marketed vaccines (Comirnaty©, Vaxzevria©, Jcovden© and Spikevax©), which included approximately 130,000 subjects in total, some adverse events (AEs) were reported more frequently in the experimental groups compared to placebo, such as pain at the injection site, redness and swelling, headache, fatigue, myalgia and arthralgia, malaise, nausea, vomiting, lymphadenopathy and fever [14,15,16,17]. These AEs are mild and commonly associated with the majority of vaccines currently on the market.

Regarding serious adverse events (SAEs), no significant differences were observed in the overall percentage reported between the experimental groups and the placebo group. Nonetheless, if the reported SAEs are studied individually, some imbalances emerge for certain preferred terms for the system organ class (SOC) “nervous system”. Specifically, certain neurological events were reported more frequently with some COVID-19 vaccines, including peripheral facial paralysis, transverse myelitis, multiple sclerosis, Guillain–Barré syndrome and seizures. Additionally, imbalances were noted for other SAEs like venous thromboembolism and embolic stroke [14,15,16,17]. It should be noted that the results of these safety analyses performed prior to the commercialization of the vaccines included data from patients with an average follow-up of 2 months after having received the complete vaccination regimen. Consequently, these pre-commercialization studies may not have detected adverse effects with longer latency times.

As wider vaccination campaigns progressed, post-marketing surveillance revealed additional adverse effects, further confirming the link between specific vaccines and certain rare events initially identified in clinical trials. Some noteworthy adverse events requiring close monitoring include the following: anaphylaxis [18], myocarditis and pericarditis (both have been observed in very rare cases—less than 1 in 10,000 people vaccinated—with Comirnaty©, Spikevax©, Jcovden© and Nuvaxovid©, usually within 14 days of the second vaccination in younger males) [19,20], thrombosis with thrombocytopenia syndrome (TTS) (around 1000 cases of TTS have been reported following vaccination with Vaxzevria© and Jcovden©) [21] and Guillain–Barré syndrome (it has been reported as a very rare side effect—less than 1 case in 10,000 people vaccinated—with the COVID-19 vaccines Jcovden© and Vaxzevria©) [12]. Even after nearly four years of widespread use, the relationship between COVID-19 vaccines and certain adverse events, such as transverse myelitis, aseptic meningitis and peripheral facial paralysis, remains under investigation. This underscores the critical importance of continuous pharmacovigilance efforts to ensure vaccine safety and inform public health recommendations.

### 1.2. Vaccine Pharmacovigilance

Pharmacovigilance, the systematic monitoring of adverse events following exposure to pharmaceutical products, is a critical component in ensuring the safety and efficacy of drugs, vaccines and other medicinal products [22]. While randomized clinical trials are widely considered the gold standard for evaluating the safety of novel pharmaceuticals, their design often includes small, homogeneous populations observed over short duration periods, limiting their capacity to detect a broader spectrum of AEs. For this reason, post-market surveillance plays a crucial role in detecting and reporting suspected AEs encountered in routine clinical practice [23].

Different approaches have been proposed to effectively detect and report AEs. One widely used approach involves causality algorithms, which offer a straightforward and consistent method for assessing AEs. However, their predictive value varies; some algorithms are tailored to specific nosological entities, while others employ a one-size-fits-all approach that can overly limit therapeutic options [24].

Another strategy involves quantifying reporting rates from both healthcare providers and patients [25]. These individual reports are crucial because they provide invaluable real-world data, enabling the identification of rare or delayed AEs and ultimately contributing to a more comprehensive understanding of medication safety. This is particularly true for large-scale databases like EudraVigilance (European Union Drug Regulating Authorities Pharmacovigilance), which plays a vital role in pharmacovigilance at the European level.

### 1.3. EudraVigilance

EudraVigilance is the system for managing and analyzing information on suspected adverse effects of medicines that have been authorized or are being studied in clinical trials in the European Economic Area [26]. The information collected is compiled in an electronic database of public access, which enhances the safe and effective use of medicines by enabling the following:

- The electronic sharing of individual case safety reports (ICSRs) among the EMA, national competent authorities, marketing authorization holders and clinical trial sponsors within the European Economic Area.
- The early identification and assessment of potential safety signals.
- Improved product information for medicines approved in the European Economic Area.

The main objective of EudraVigilance is the early detection of possible safety signals from marketed drugs for human use through continuous monitoring and evaluation of potential safety issues in relation to reported adverse effects. The European Medicines Agency (EMA) and national competent authorities are responsible for regularly reviewing and analyzing EudraVigilance data to detect safety signals. The Pharmacovigilance Risk Assessment Committee evaluates the safety signals detected in EudraVigilance and may recommend regulatory action as a result. EMA also publishes annual reports to provide a summary of the EudraVigilance-related activities the Agency undertakes within the European medicines regulatory network and with stakeholders [26]. EudraVigilance data are publicly accessible, enabling the conduct of observational studies, including disproportionality analysis, to investigate potential links between adverse effects and specific medications.

### 1.4. Disproportionallity Analyses

Disproportionality analyses are observational studies that constitute valid methods for the generation of hypotheses aimed at detecting “safety signals”. Safety signals are possible existing relationships between a drug or medical device and an adverse effect generated through the analysis of information extracted from databases containing reports of possible adverse events. By quantifying the degree to which a drug–adverse effect pair occurs disproportionately (i.e., with a higher frequency than would be expected if there was no relationship between that drug and the development of the AE under study), it may be hypothesized that the adverse effect is indeed causally related to the study drug [27].

It should be noted that the real number of patients exposed to the drug being studied is unknown, since only those cases that have been reported in the database as possible adverse effects are taken into account. Therefore, disproportionality analyses cannot quantify or estimate the real risk between a drug and an AE, and they do not allow causality to be inferred, their sole purpose being to provide a knowledge base on which to subsequently develop analyses with more robust methodologies that do allow the generated hypothesis to be further investigated [28].

One of the statistical methods used to try to investigate the possible relation between an adverse effect and a drug in disproportionality studies is the Proportional Reporting Ratio (PRR), which is the method used in the present study.

### 1.5. Rationale and Objectives of This Study

Since the commercialization of COVID-19 vaccines, several cases of various neurological entities in patients with no relevant medical history exposed to different COVID-19 vaccines have been published in the literature [29,30,31,32]. Similarly, in September 2021, a case of aseptic meningitis after the administration of the first dose of Comirnaty© was detected through the Hospital Pharmacovigilance Program by Laboratory Signals (PFVHSL for its acronym in Spanish) and reported by our Pharmacovigilance Unit in the La Paz University Hospital in Madrid, Spain [33]. In the immediate aftermath of the COVID-19 outbreak, as the SARS-CoV-2 vaccine rollout reached its peak, pharmacovigilance schemes garnered heightened significance due to concerns from healthcare professionals and public skepticism. This led to an unprecedented increase in both the detection and reporting of vaccine-related AEs in an effort to determine whether the benefits outweighed the risks, ultimately shaping public healthcare policy [34].

The objectives of this study are two-fold:

Firstly, we evaluated potential safety signals linking COVID-19 vaccine administration to several neurological adverse effects (aseptic meningitis, Guillain–Barré syndrome, polyradiculoneuropathies, multiple sclerosis, transverse myelitis and neuromyelitis optica) using disproportionality analysis of available EudraVigilance reports. This analysis aims to identify signals warranting further investigation with robust methodologies.

Secondarily, given the high proportion of adverse events reports from non-healthcare professionals, particularly during the initial phases of vaccine rollout, we evaluated the influence of the reporting source on disproportionality study results. We hypothesize that reports from non-health professionals, often concerning mild adverse events, may dilute the signal for serious and specific AEs by increasing the denominator in PRR calculations. This analysis will help evaluate the potential impact of the reporting source on pharmacovigilance data interpretation.

## 2. Results

**Figure 1:**
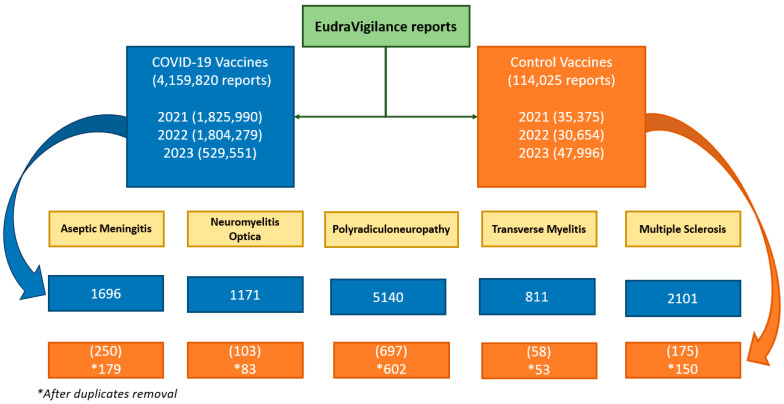
Flowchart.

A total of 4,159,820 reports for COVID-19 vaccines and 114,025 for the 47 comparator vaccines were compiled. A decline in the number of reports submitted to EudraVigilance can be observed over the years, with a particularly notable drop in 2023. A total of 4,159,820 reports for COVID-19 vaccines and 114,025 for the 47 comparator vaccines were compiled. In 2021 1,825,990 reports for COVID-19 vaccines and 35,375 reports for the comparator vaccines were registered; whereas in 2022, the numbers were 1,804,279 and 30,654, respectively, and in 2023, they were 529,551 and 47,996, respectively (Figure 1).

Of the reports collected for the control vaccines: 71, 20, 95, 5 and 25 duplicate cases were excluded. Figure 1 shows the flowchart of neurological AEs reported following COVID-19 vaccinations and comparator vaccinations in EudraVigilance.

**Figure 2:**
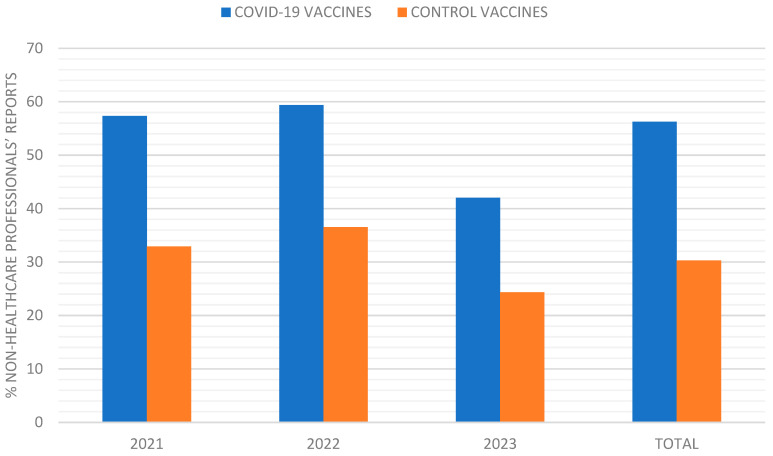
Evolution of the % of non-healthcare professionals’ reports from 2021 to 2023.

The percentage of AE reports submitted by non-healthcare professionals was consistently higher for COVID-19 vaccines compared to control vaccines. In 2021, 57.33% of COVID-19 vaccine reports were from non-healthcare professionals, compared to 33% for control vaccines (*p* < 0.001). This trend continued in 2022 (59.4% vs. 36.5%, *p* = 0.001) and 2023 (42% vs. 24.36%, *p* = 0.006) (Figure 2).

### 2.1. Primary Objective: Relationship Between COVID-19 Vaccines and Neurological Adverse Effects

To evaluate the possible relationship between the COVID-19 vaccines and the different neurological adverse effects under study, we calculated the PRR. We examined each vaccine separately and then combined to assess overall trends. Since EudraVigilance data are organized by calendar year, we calculated PRRs for 2021, 2022 and 2023 individually and then for the combined period from January 2021 to December 2023, allowing us to track any changes in reporting trends over time.

#### 2.1.1. 2021

In 2021, none of the neurological adverse effects obtained a PRR ≥ 2, the threshold necessary to suggest a potential neurological safety signal. The only vaccine that obtained a PRR significantly higher than 1 was Jcovden© for polyradiculoneuropathy with a PRR of 1.52 (IC 95% 1.29–1.80). It is important to note that while Nuvaxovid© was authorized in the EU during this period, no adverse effects associated with it were reported in EudraVigilance, resulting in a PRR of 0 for all neurological conditions (Table 1).

#### 2.1.2. 2022

Similarly to 2021, in 2022, no vaccine achieved a PRR ≥ 2. Notably, adverse effects associated with Nuvoxavid© were reported in 2022, as shown in Table 2, although not for all the neurological conditions under investigation.

#### 2.1.3. 2023

In 2023, several significant safety signals (PRR ≥ 2) emerged between specific COVID-19 vaccines and neurological adverse effects. Jcovden© exceeded the significance threshold for polyradiculoneuropathy [PRR 5.4, IC 95% 3.98–7.32)], multiple sclerosis [PRR 2.72, IC 95% (1.08–6.87)], transverse myelitis [PRR 4.68, IC 95% (1.02–21.35)] and neuromyelitis optica [PRR 7.79, IC 95% (3.5–17.37)]. In addition, both Spikevax© and Comirnaty© showed significant safety signals with multiple sclerosis [PRR 2.50, IC 95% (1.70–3.68), and PRR 2.33, IC 95% (1.68–3.24), respectively] and transverse myelitis [PRR 3.50, IC 95% (1.66–7.50) and PRR 3.58, IC 95% (1.85–6.93), respectively] (Table 3). These findings highlight a potential increased risk of these neurological events following vaccination with these specific vaccines and warrant further investigation.

#### 2.1.4. Total Period (January 2021–December 2023)

Despite the significant signals observed in 2023, an analysis of the combined three-year dataset revealed no vaccine with a PRR exceeding the threshold of 2 (Table 4). This is likely due to the higher number of cases reported in 2021 and 2022, which heavily influenced the overall PRR calculations, diluting the impact of the 2023 findings. Essentially, the larger volume of data from the earlier years, where no significant signals were found, pulled the overall PRR values down.

### 2.2. Secondary Objective: Relationship Between Percentage of Events Reported by Non-Healthcare Professionals and Proportional Reporting Ratio

Given the high proportion of adverse effects reported by non-healthcare professionals and the low PRRs observed, we investigated whether the reporting source influenced the PRR values. Specifically, we examined the relationship between the percentage of reports from non-healthcare professionals and the calculated PRRs.

To assess if reports from non-healthcare professionals might obscure potential links between vaccines and specific adverse effects, we used Spearman’s correlation coefficient (SCC; ρ). This analysis compared the percentage of non-healthcare professional reports with the case/no-case ratio (calculated by dividing the number of each specific neurological adverse event by the total number of adverse events) for each neurological adverse effect associated with both COVID-19 vaccines and control vaccines across 2021, 2022 and 2023 (Table 5).

With the 30 paired observations, the calculation of SCC was −0.4683 (*p* = 0.009), which indicates a moderate negative correlation between the two variables being analyzed. This suggests that a higher percentage of reports from non-healthcare professionals tends to be associated with lower PRR values. While this relationship is not extremely strong, it is sufficient to highlight a notable trend between non-healthcare professional (non-HCP) reports and the Proportional Reporting Ratio (PRR). This finding suggests that an increased volume of non-healthcare professional reports could potentially dilute the PRR, potentially impacting the reliability of signals in pharmacovigilance systems. This emphasizes the need for careful interpretation of signals in systems with a high proportion of non-healthcare professional contributions, as these reports might differ in content or accuracy compared to those from healthcare professionals. However, this relationship is not perfectly linear, indicating that other factors likely contribute to this trend. Further investigation is needed to understand the complex interplay between the reporting source and PRR calculations.

## 3. Discussion

After examining the results of the PRR calculation of 4,159,820 COVID-19 vaccine reports and 114,025 comparator vaccine reports from EudraVigilance, the only vaccine with a PRR greater than 2 was Jcovden© in relation to polyradiculoneuropathy and neuromyelitis optica, and only in the analysis of the 2023 data. Notably, no significant PRR was observed for any COVID-19 vaccine across the three-year period when considering all neurological effects under study.

Since the marketing authorization of the COVID-19 vaccines, polyradiculoneuropathies such as Guillain–Barré have already been described and appear in the summary of product characteristics (SmPCs) for both Jcovden© (as seen in the 2023 results of this study) and Vaxzevria©; and despite not appearing in the SmPCs of any of the COVID-19 vaccines, cases of optic neuritis have been described in the literature in previously healthy persons vaccinated with Comirnaty©, Spikevax© and Jcovden© [35] (as also shown in the 2023 results of the present study). However, it is striking that in this disproportionality study, no high PRRs were obtained for any of the COVID-19 vaccines for transverse myelitis, when it is an effect that has been described in the literature for almost all COVID-19 vaccines [36] and already appears in the Jcovden© and Vaxzevria© SmPCs. These results are probably related to the notable fact that in these three years of data collection, almost 98% of the reports collected were for COVID-19 vaccines (4,159,820), and of these, 56.28% were made by non-healthcare professionals, compared to the 30.29% of reports made by non-healthcare professionals for control vaccines (Figure 1 and Figure 2). This phenomenon likely reflects the extensive vaccination campaigns’ widespread information dissemination regarding potential vaccination risks and heightened public health awareness during the pandemic, all contributing to an unprecedented influx of adverse event reports. This influx may have diluted the signal for specific neurological events within the large volume of reported data.

A comprehensive analysis of adverse event reports reveals a predominance of mild effects associated with COVID-19 vaccines, typically characterized by post-vaccination symptoms such as fever, injection site pain, myalgia, and headache. Conversely, reports for comparator vaccines exhibit a higher proportion of moderate to severe adverse effects. This disparity may be attributed to the lower percentage of reports submitted by non-healthcare professionals for comparator vaccines or their longer market presence, leading to a large denominator in PRR calculations and a reduced case/no-case ratio for COVID-19 vaccines. This reduction may obscure potential signals between specific adverse effects and COVID-19 vaccines. These findings are consistent with the data obtained in this study. For example, in 2023, an increase in the PRR is observed across almost all COVID-19 vaccines, coinciding with a decrease in the percentage of AEs reported by non-healthcare professionals.

The findings observed from the analysis of EudraVigilance in the present study align with trends observed in VAERS and the Yellow Card scheme. Both systems have reported a predominance of mild adverse effects for COVID-19 vaccines, such as fever, injection site pain and headache [37,38]. Both systems have observed that reports for comparator vaccines tend to include a higher proportion of moderate to severe adverse effects. This may be influenced by factors such as the longer market presence of comparator vaccines and differences in reporting behaviors among healthcare professionals and the general public [39,40].

When examining the trends in both the total number and percentage of AEs reported by non-healthcare professionals and comparing them with the evolution of the PRRs, a certain signal is observed. This signal is further supported by the calculated Spearman’s correlation coefficient, which, with a result of −0.4683 and a *p*-value of 0.009, indicates an inverse relationship between these two variables. Nonetheless, it is important to remember that correlation does not imply causation; thus, while the two variables are related, further investigation using additional statistical tests or data exploration is necessary to understand the underlying dynamics and potential causal factors.

### Strengths and Limitations

The present disproportionality study has a robust information base, as the reports that have been collected for the analysis come from EudraVigilance, which has a very large number of reports from all countries in the European Economic Area. Therefore, its strength comes from its ability to harness a vast amount of data from over a million reports, which enhances its statistical power and robustness. This extensive dataset can allow us to identify potential safety signals and patterns that may not be evident in smaller studies. Additionally, the large sample size employed increases the likelihood of capturing rare adverse events, providing a more comprehensive understanding of the relationship between medications and reported outcomes.

On the other hand, while leveraging a wide dataset of over a million reports and thus possessing great statistical power, this study comes with inherent limitations characteristic of observational studies. Despite its ability to identify potential signals between variables, it cannot establish causality; observed correlations may be influenced by confounding factors or biases inherent in the reporting system. Additionally, the analysis relies heavily on the accuracy and completeness of the data collected, which can vary significantly across reports. Variability in reporting practices, differences in the population studied, and the potential for underreporting or overreporting of certain events can further skew results, which has possibly occurred given the reporting differences between COVID-19 vaccines and the rest of the vaccines in the studied period. This suggests that reports made by non-health professionals are not as accurate as those made by health professionals, who also tend to report more complicated and therefore more serious nosological entities. Moreover, another limitation of using a database such as EudraVigilance is that a unique report may contain multiple preferred terms describing the same AE. The search would therefore return several results for the same adverse event reported, resulting in an overestimation of some adverse events. There is no simple way to solve this problem, since, as explained above, the sheer number of reports makes it impossible to discard possibly duplicate reports. Consequently, while the findings can offer valuable insights, they should be interpreted with caution and complemented by further research to validate the signals identified.

## 4. Materials and Methods

### 4.1. Study Design

**Figure 3:**
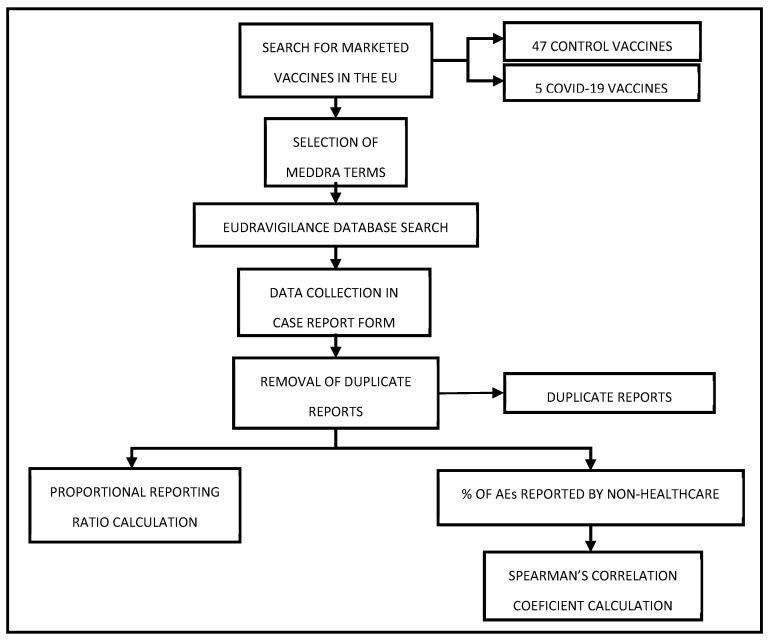
Work Scheme.

In order to detect potential safety signals linking COVID-19 vaccines and the development of the aforementioned neurological adverse effects, we conducted a disproportionality analysis using EudraVigilance data from January 2021 to December 2023. The search and data compilation were performed in August 2024. Figure 3 provides a visual representation of this study’s work plan.

### 4.2. Data Source and Data Mining

First, we compiled all vaccines marketed in the European Union (EU) and the European Economic Area so that they could be used as a comparator against the COVID-19 vaccines that had a significant amount of reports in EudraVigilance at the time of data compilation (Comirnaty©, Vaxzevria©, Jcovden©, Spikevax© and Nuvaxovid©). A significant number of vaccines are marketed within the European Economic Area. Some of these vaccines are available only in specific countries, while others are commercialized in various combinations or targeting different viral serotypes. For the purposes of the analysis, it was decided to include reports for all vaccines with at least one notification of an adverse reaction in EudraVigilance. The 47 marketed vaccines (as categorized in EudraVigilance) that were finally used as comparators can be found in Table 6. These vaccines were as follows: rotavirus (monovalent and pentavalent); tetanus; diphtheria; diphtheria + tetanus; diphtheria + tetanus + hepatitis B virus (HBV); diphtheria + tetanus + poliomyelitis; diphtheria + tetanus + pertussis; diphtheria + tetanus + pertussis + *Haemophilus influenzae* b (Hib); diphtheria + tetanus + pertussis + HBV; diphtheria + tetanus + pertussis + HBV + Hib; diphtheria + tetanus + pertussis + poliomyelitis; diphtheria + tetanus + pertussis + poliomyelitis + Hib; diphtheria + tetanus + pertussis + poliomyelitis + HBV; diphtheria + tetanus + pertussis + poliomyelitis + Hib + HBV; poliomyelitis (Salk and Sabin); Hib; Hib + HBV; Hib + meningitis C (MenC); HBV; HBV + hepatitis A virus (HAV); HAV + typhoid fever; pneumococcus; meningitis A (MenA); MenC; meningitis B (MenB); rubella; chickenpox; measles; measles + rubella; measles + rubella + mumps; measles + rubella + mumps + chickenpox; human papillomavirus (bivalent, tetravalent and nonavalent); influenza; herpes zoster; tick-borne virus; tuberculosis and rabies.

Then, a systematic search of the neurological effects studied (aseptic meningitis, polyradiculoneuropathies, multiple sclerosis, transverse myelitis and neuromyelitis optica) was conducted. This search encompassed each of the 5 COVID-19 vaccines and used the 47 non-vaccines as comparators, spanning three distinct periods: January 2021–December 2021, January 2022–December 2022 and January 2023–December 2023. EudraVigilance utilizes MedDRA terminology to classify adverse effect reports. To identify the neurological effects under investigation, we used the following MedDRA terms:

- Aseptic meningitis: “Meningeal disorder”; “meningeal thickening”; “meningism”; “meningitis”; “meningitis aseptic”; “meningitis eosinophilic”; “meningitis noninfective” and “meningoradiculitis”.
- Polyradiculoneuropathies: “polyradiculoneuropathy”; “Guillain-Barre syndrome”; “acute polyneuropathy”; “ascending flaccid paralysis” and “chronic inflammatory demyelinating polyradiculoneuropathy”.
- Multiple sclerosis: “multiple sclerosis”; “multiple sclerosis relapse” and “multiple sclerosis pseudo relapse”.
- Transverse myelitis: “Myelitis transverse”.
- Neuromyelitis optica: “neuromyelitis optica spectrum disorder”; “neuromyelitis optica psuedo relapse”; “optic nerve disorder”; “optic neuritis”; “optic neuropathy”; “optic perineuritis”.

**Figure 4:**
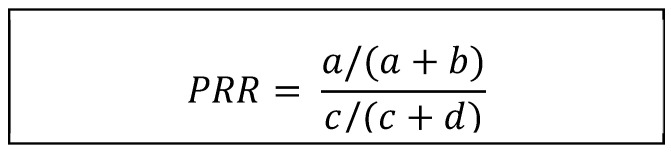
Proportional Reporting Ratio calculation.

The EudraVigilance reports from January 2021 to December 2023 were analyzed, quantifying neurological adverse effects for each of the 52 vaccines included in this study (5 COVID-19 vaccines and 47 comparators). The data were compiled and analyzed using Microsoft Excel 2013^®^. While we attempted to eliminate duplicate reports using EudraVigilance reporting codes, this was not feasible for COVID-19-vaccine-related neurological adverse effects due to the high volume of reports. This fact may have influenced the calculation of the Proportional Reporting Ratio, increasing the potential safety signals linking COVID-19 vaccine administration to several neurological adverse effects by increasing the numerator of the calculation (Figure 4).

**Figure A1:**
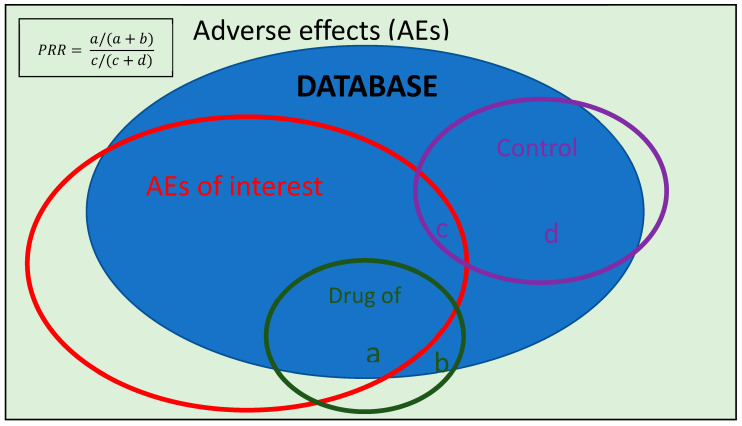
Proportional Reporting Ratio’s calculation explanation.

Here, “a” is the number of neurological adverse effects reported for the medicinal product under investigation (COVID-19 vaccines), “b” is the total AEs reported for that product, “c” is the number of neurological AEs reported for the medicinal products used as comparators (the non-COVID-19 marketed vaccines in the EEA) and “d” is the total adverse effects reported for the comparators. An explanatory figure can be found in Appendix A, Figure A1.

In addition to the formal calculation of the PRR, 95% confidence interval (CI 95%) values were calculated for each of the calculated PRRs. A PRR of 2 or higher, with a lower CI limit greater than 1, suggests a statistically significant neurological safety signal warranting further investigation with more robust methods.

Additionally, we analyzed the potential influence of the reporting source on disproportionality analysis. We calculated the percentage of AE reports submitted by non-healthcare professionals for each of the vaccines and each neurological AEs. To test the hypothesis that reports from non-healthcare professionals may obscure the true relationship between serious and specific adverse effects, we calculated Spearman’s correlation coefficient (SCC, ρ), comparing the percentage of non-healthcare professionals’ reports with the case/no-case ratio for each of the neurological adverse effects with both the COVID-19 vaccines and the control vaccines in 2021, 2022 and 2023. All data analysis was performed using RStudio V 4.3.1 (Integrated Development for R. RStudio, PBC, Boston, MA; http://www.rstudio.com, accessed on 22 October 2024).

## 5. Conclusions

While a PRR exceeding 2 was observed only for Jcovden©, Spikevax© and Comirnaty© in 2023, with no vaccines exceeding this threshold in the combined three-year analysis, this may be attributed to the high proportion of AE reports submitted by non-healthcare professionals for COVID-19 vaccines in 2021 and 2022. This is supported by the observed inverse correlation between the percentage of reports from non-healthcare professionals and the case/no-case ratio. Future replications with more homogeneous data may yield more accurate results.

This finding underscores the critical importance of high-quality, homogeneous data for robust PRR estimation, particularly for rare adverse events such as neurological events. Moreover, despite this study’s obvious limitations, its low-cost and straightforward methodology makes it very cost-effective, enabling researchers to quickly analyze trends and generate hypotheses for further investigation.

Ultimately, this study emphasizes the need for continuous improvement in data quality within pharmacovigilance systems, like Eudravigilance. By enhancing data quality and addressing potential biases, disproportionality analyses can serve as a valuable first step in identifying potential safety signals and guiding more in-depth research to optimize public health outcomes.

## Figures and Tables

**Table 1:** Proportional Reporting Ratio of COVID-19 vaccines in 2021.

	Proportional Reporting Ratio (95% CI) 2021				
	Aseptic Meningitis	Polyradiculoneuropathy	Multiple Sclerosis	Transverse Myelitis	Neuromyelitis Optica
**SARS-CoV-2 vaccines**	0.16 (0.12–0.22)	0.25 (0.22–0.29)	0.30 (0.23–0.40)	0.30 (0.20–0.45)	0.32 (0.23–0.46)
**Spikevax (Moderna)**	0.35 (0.25–0.48)	0.33 (0.28–0.39)	0.54 (0.40–0.72)	0.67 (0.43–1.04)	0.54 (0.36–0.80)
**Comirnaty (Pfizer-BioNTech)**	0.25 (0.19–0.34)	0.22 (0.19–0.26)	0.49 (0.37–0.64)	0.28 (0.18–0.43)	0.48 (0.34–0.69)
**Vaxzevria (AstraZeneca)**	0.07 (0.05–0.10)	0.20 (0.17–0.23)	0.13 (0.10–0.17)	0.20 (0.13–0.31)	0.17 (0.11–0.24)
**Jcovden (Janssen)**	0.20 (0.11–0.36)	1.52 (1.29–1.80)	0.33 (0.21–0.54)	1.33 (0.80–2.20)	0.70 (0.43–1.16)
Nuvaxovid (Novavax) *	0	0	0	0	0

* At the time of data download, Nuvaxovid© was authorized in the European Union, but no adverse effect had been reported with Nuvaxovid© as a suspected medication in EudraVigilance.

**Table 2:** Proportional Reporting Ratio of COVID-19 vaccines in 2022.

	Proportional Reporting Ratio (95% CI) 2022				
	Aseptic Meningitis	Polyradiculoneuropathy	Multiple Sclerosis	Transverse Myelitis	Neuromyelitis Optica
**SARS-CoV-2 vaccines**	0.26 (0.19–0.34)	0.18 (0.16–0.22)	0.31 (0.23–0.42)	0.27 (0.17–0.44)	0.30 (0.20–0.45)
**Spikevax (Moderna)**	0.21 (0.14–0.31)	0.25 (0.21–0.31)	0.59 (0.43–0.81)	0.32 (0.18–0.56)	0.55 (0.35–0.86)
**Comirnaty (Pfizer-BioNTech)**	0.23 (0.16–0.31)	0.29 (0.24–0.34)	0.69 (0.52–0.93)	0.42 (0.26–0.68)	0.62 (0.41–0.94)
**Vaxzevria (AstraZeneca)**	0.28 (0.21–0.38)	0.09 (0.07–0.11)	0.05 (0.04–0.07)	0.16 (0.10–0.26)	0.08 (0.05–0.12)
**Jcovden (Janssen)**	0.13 (0.05–0.30)	1.11 (0.89–1.38)	0.46 (0.27–0.76)	1.27 (0.68–2.38)	0.38 (0.18–0.80)
**Nuvaxovid (Novavax)**	0.42 (0.06–3.10)	0.26 (0.07–1.06)	0.87 (0.21–3.58)	0	0

**Table 3:** Proportional Reporting Ratio of COVID-19 vaccines in 2023.

	Proportional Reporting Ratio (95% CI) 2023				
	Aseptic Meningitis	Polyradiculoneuropathy	Multiple Sclerosis	Transverse Myelitis	Neuromyelitis Optica
**SARS-CoV-2 vaccines**	0.58 (0.45–0.74)	0.24 (0.20–0.28)	0.62 (0.45–0.86)	1.19 (0.62–2.26)	0.59 (0.38–0.91)
**Spikevax (Moderna)**	0.48 (0.30–0.79)	0.62 (0.48–0.80)	2.50 (1.70–3.68)	3.50 (1.66–7.50)	1.96 (1.14–3.39)
**Comirnaty (Pfizer-BioNTech)**	0.37 (0.26–0.52)	0.73 (0.62–0.87)	2.33 (1.68–3.24)	3.58 (1.85–6.93)	1.95 (1.24–3.05)
**Vaxzevria (AstraZeneca)**	0.63 (0.49–0.81)	0.07 (0.05–0.08)	0.07 (0.04–0.11)	0.43 (0.21–0.87)	0.13 (0.07–0.23)
**Jcovden (Janssen)**	1.92 (0.83–4.42)	5.40 (3.98–7.32)	2.72 (1.08–6.87)	4.68 (1.02–21.35)	7.79 (3.50–17.37)
**Nuvaxovid (Novavax)**	0	0.60 (0.08–4.33)	0	0	0

**Table 4:** Proportional Reporting Ratio of COVID-19 vaccines from 2021.

	Proportional Reporting Ratio (95% CI) 2021–2023				
	Aseptic Meningitis	Polyradiculoneuropathy	Multiple Sclerosis	Transverse Myelitis	Neuromyelitis Optica
**SARS-CoV-2 vaccines**	0.26 (0.22–0.30)	0.23 (0.22–0.25)	0.38 (0.33–0.45)	0.42 (0.32–0.55)	0.39 (0.31–0.48)
**Spikevax (Moderna)**	0.3 (0.24–0.37)	0.33 (0.30–0.37)	0.76 (0.63–0.92)	0.77 (0.56–1.05)	0.71 (0.55–0.92)
**Comirnaty (Pfizer-BioNTech)**	0.25 (0.21–0.30)	0.30 (0.27–0.33)	0.78 (0.66–0.92)	0.56 (0.42–0.74)	0.72 (0.55–0.90)
**Vaxzevria (AstraZeneca)**	0.26 (0.22–0.30)	0.14 (0.13–0.16)	0.10 (0.08–0.12)	0.24 (0.18–0.33)	0.14 (0.11–0.18)
**Jcovden (Janssen)**	0.22 (0.14–0.33)	1.58 (1.41–1.77)	0.51 (0.37–0.71)	1.86 (0.29–2.68)	0.85 (0.59–1.22)
**Nuvaxovid (Novavax)**	0.35 (0.05–2.50)	0.31 (0.10–0.97)	0.83 (0.21–3.36)	0	0

**Table 5:** Spearman’s correlation coefficient of the % of non-healthcare professionals’ reports and the case/no-case ratio.

		% of Non-Healthcare Professionals’ Reports	Case/No-Case Ratio
**Aseptic meningitis**	**Control vaccines 2021**	32.91	0.1611
	**Control vaccines 2022**	36.55	0.1600
	**Control vaccines 2023**	24.36	0.1522
	**COVID-19 vaccines 2021**	57.34	0.0264
	**COVID-19 vaccines 2022**	59.39	0.0408
	**COVID-19 vaccines 2023**	42.02	0.0886
**Polyradiculoneuropathy**	**Control vaccines 2021**	32.91	0.6163
	**Control vaccines 2022**	36.55	0.5160
	**Control vaccines 2023**	24.36	0.4693
	**COVID-19 vaccines 2021**	57.34	0.1551
	**COVID-19 vaccines 2022**	59.39	0.0948
	**COVID-19 vaccines 2023**	42.02	0.1122
**Multiple sclerosis**	**Control vaccines 2021**	32.91	0.1668
	**Control vaccines 2022**	36.55	0.1566
	**Control vaccines 2023**	24.36	0.0896
	**COVID-19 vaccines 2021**	57.34	0.0504
	**COVID-19 vaccines 2022**	59.39	0.0491
	**COVID-19 vaccines 2023**	42.02	0.0559
**Transverse myelitis**	**Control vaccines 2021**	32.91	0.0707
	**Control vaccines 2022**	36.545	0.0587
	**Control vaccines 2023**	24.36	0.0208
	**COVID-19 vaccines 2021**	57.34	0.0214
	**COVID-19 vaccines 2022**	59.39	0.0160
	**COVID-19 vaccines 2023**	42.02	0.0247
**Neuromyelitis optica**	**Control vaccines 2021**	32.91	0.0962
	**Control vaccines 2022**	36.55	0.0816
	**Control vaccines 2023**	24.36	0.0500
	**COVID-19 vaccines 2021**	57.34	0.0314
	**COVID-19 vaccines 2022**	59.39	0.0244
	**COVID-19 vaccines 2023**	42.02	0.0295
	**SCC (** **ρ)**	−0.4683	*p* = 0.009

**Table 6:** Marketed non-COVID-19 vaccines in the EEA as categorized in EudraVigilance.

Vaccines
**Rotavirus**
**Tetanus**
**Diphtheria**
**Diphtheria + tetanus**
**Diphtheria + tetanus + hepatitis B virus (HBV)**
**Diphtheria + tetanus + poliomyelitis**
**Diphtheria + tetanus + pertussis**
**Diphtheria + tetanus + pertussis + *Haemophilus influenzae* b (Hib)**
**Diphtheria + tetanus + pertussis + HBV**
**Diphtheria + tetanus + pertussis + poliomyelitis**
**Diphtheria + tetanus + pertussis + poliomyelitis** **+ Hib**
**Diphtheria + tetanus + pertussis + poliomyelitis + HBV**
**Diphtheria + tetanus + pertussis + poliomyelitis** **+ HBV + Hib**
**Diphtheria + tetanus + pertussis** **+ HBV + Hib**
**Poliomyelitis**
**Hib**
**Hib + HBV**
**Hib + Meningitis C (MenC)**
**HBV**
**HBV + hepatitis A virus (HAV)**
**HAV**
**HAV +** **typhoid fever**
**Pneumococcus**
**MenA, C, W135, Y**
**MenB**
**MenC**
**Rubella**
**Chickenpox**
**Chickenpox + measles**
**Measles + rubella**
**Measles + rubella + mumps**
**Measles + rubella + mumps + chickenpox**
**Human papillomavirus (HPV)**
**Influenza**
**Herpes zoster**
**Tick-borne virus**
**Tuberculosis**
**Rabies**

## Data Availability

The data supporting the findings of this study are publicly available from the EudraVigilance database. Access to and downloading of these data are available to the public via the EudraVigilance website.

## Abbreviations

The following abbreviations are used in this manuscript:

AE	Adverse effect
SAE	Serious adverse event
EEA	European Economic Area
EMA	European Medicines Agency
PRR	Proportional Reporting Ratio
